# The Role of Reactive Oxygen Species (ROS) in Periodontitis: A Potential Therapeutic Target

**DOI:** 10.1002/iid3.70301

**Published:** 2025-11-26

**Authors:** Shuyu Yang, Xi Yang

**Affiliations:** ^1^ Stomatological Hospital, School of Stomatology, Southern Medical University Guangzhou China

**Keywords:** antioxidants, MAPK, NF‐κB, NLRP3, PDT, periodontitis, reactive oxygen species

## Abstract

**Background:**

ROS are pivotal in maintaining periodontal tissue health due to their dual role in physiological and pathological states. At physiological levels, ROS are essential for host defense, effectively eliminating pathogenic bacteria during inflammation and sustaining the homeostasis of the periodontal microenvironment. However, excessive ROS production disrupts the balance between oxidative activity and antioxidant defenses, leading to oxidative stress (OS). This imbalance exacerbates inflammation, damages cellular components, and drives periodontal tissue destruction. Recognizing the dual role of ROS underscores the importance of studying their regulatory mechanisms, which is crucial for advancing understanding and therapeutic strategies in periodontitis management.

**Objective:**

This review summarizes the latest research advancements on the molecular mechanisms linking ROS to the progression of periodontitis and explores related therapeutic strategies.

**Results:**

This review focuses on the pivotal role and specific mechanisms of periodontal tissue destruction by excess ROS, which mediate signaling pathways such as nuclear factor kappa B (NF‐κB) and mitogen‐activated protein kinase (MAPK), as well as the activation of OS response‐associated proteins, including thioredoxin‐interacting protein (TXNIP) and mitochondrial antiviral signaling protein (MAVS). Additionally, we provide a concise summary of emerging therapeutic strategies for periodontitis, including the use of antioxidants, photodynamic therapy (PDT), and nanomaterials. Gaining a more profound comprehension of these mechanisms may pave the way for the formulation of enhanced therapeutic strategies for periodontitis.

**Conclusion:**

Understanding the interplay between ROS and periodontal tissue destruction is essential for advancing periodontitis research. Targeting the NOD‐like receptor family pyrin domain‐containing 3 (NLRP3) inflammasome with antioxidant and ROS‐modulating therapies presents a promising strategy to mitigate both inflammation and OS, thereby reducing periodontal degradation.

## 1 Introduction

Periodontitis ranks among the most widespread inflammatory conditions, which affects nearly half of the global population [1]. Clinically, it manifests as gingival bleeding, formation of periodontal pockets, alveolar bone loss, and, in severe cases, tooth loss [2]. The primary pathogen behind the inflammatory lesions in periodontal tissues is subgingival dental plaque biofilm, which mainly consists of Gram‐negative anaerobic or facultative anaerobic pathogens, such as *Porphyromonas gingivalis (P. gingivalis)*, *Aggregatibacter actinomycetemcomitans (Aa)*, and *Fusobacterium nucleatum (Fn)* [3]. Polymorphonuclear leukocytes (PMNs) and macrophages, key immune players, release antimicrobial substances, engulf bacteria, and promote an inflammatory response [4]. Neutrophils, in particular, release large amounts of ROS, which play essential roles in immune defense during pathological conditions [5]. ROS are oxygen‐containing chemical species that are generally classified into two main types [1]: free radical ROS, including superoxide anion (O2·‐), hydroxyl radical (·OH), alkoxyl (OOR), and peroxyl radical (OOH); and [2] non‐radical ROS, such as hydrogen peroxide (H_2_O_2_), singlet oxygen (O_2_), ozone (O_3_), and hypochlorite anion (OCl−) [6, 7]. The concentration of ROS is closely linked to periodontal health, and prolonged oxidative stress (OS) is a key driver in the onset and progression of periodontitis [8].

With the growing prevalence of periodontitis and its associations with systemic conditions, understanding ROS‐driven processes is critical for developing targeted treatments. Specifically, this knowledge can guide the creation of antioxidant therapies, photodynamic therapy (PDT), ROS‐modulating drugs, and innovative biomaterials designed to not only mitigate oxidative damage but also promote tissue regeneration and clinical outcomes in patients suffering from periodontitis. Therefore, this review aims to bridge the gap between fundamental research and clinical application, providing a comprehensive framework for advancing treatment strategies in periodontal disease management.

## 2 Sources of ROS in Periodontitis

ROS play an instrumental role in periodontitis progression, originating from both endogenous and external sources (Table 1). Endogenously, ROS are predominantly produced by inflammatory cells, such as neutrophils and macrophages, in response to periodontal pathogens [9]. These immune cells generate ROS to combat invading pathogens, but elevated levels of ROS levels can lead to tissue damage and exacerbate inflammation [10]. Additionally, mitochondrial dysfunction in these cells can exacerbate ROS production. Exogenous factors encompass the presence of periodontal pathogens such as *P. gingivalis*, which not only generate ROS directly but also stimulate host cells to augment ROS production [11]. Additionally, smoking and alcohol use exacerbate OS in periodontal tissues [12]. Understanding these sources of ROS is vital for developing therapeutic strategies to mitigate OS in periodontitis.

**Table 1:** Sources of ROS in periodontitis.

Source	Mechanism	Effect on periodontitis
Periodontal pathogens [16, 19, 20, 26]	*P. gingivalis*: Impair neutrophil functionality, inhibit phagocytosis, exacerbate inflammatory damage and immune evasion *Fn*: Augment key periodontal pathogens pathogenic potential *Aa*: Synthesize leukotoxin A (LtxA), which eradicates neutrophils while inducing the release of ROS from cells	Promote periodontal pathogen growth and increase ROS production
Hyper‐reactive PMNs [35, 36]	PMNs are recruited and activated in the damaged area to clear the periodontal pathogens by releasing ROS	Activated PMNs produce excessive ROS, causing oxidative stress and periodontal tissue damage
Mitochondrial dysfunction [38]	Mitochondrial dysfunction triggers an overproduction of ROS, and mitochondrial oxidative stress damage	Aggravate oxidative stress, intensifying periodontal tissue destruction
Unhealthy lifestyle factors [56, 57, 59, 60]	High‐fat and high‐protein diets: the increased amounts of electrons transferred to the electronic respiratory chain causes a sharp increase in superoxide anions production Smoking: nicotine causes elevation in the respiratory burst of PMNs, triggers the generation of ROS Alcohol: ROS are generated during all phases of alcohol metabolism	Both factors lead to increased oxidative stress and periodontal tissue damage

### 2.1 Periodontal Pathogens

Common periodontal pathogens encompass *P. gingivalis*, *Aa*, and *Fn*. These microorganisms, which reside in the oral cavity, are pivotal in the etiology and progression of periodontitis [13]. These microorganisms persist on tooth surfaces, in the gingival epithelium, and within the oral cavity, gradually forming complex dental plaque biofilms [14]. The relationship between periodontal pathogens and ROS is intricate. Periodontal pathogens induce mitochondrial dysfunction, leading to an upregulation in ROS production [15]. ROS, in turn, influence plaque biofilm composition, establishing an environment that fosters the proliferation of pathogenic bacteria and enhances their virulence [16].

*P. gingivalis*, frequently found in the subgingival plaque of periodontitis patients, produces numerous virulence factors, including lipopolysaccharides (LPS), proteases, and fimbriae [17]. LPS activates NADPH oxidase in neutrophils and macrophages, leading to elevated ROS production and the release of pro‐inflammatory cytokines, such as interleukin‐1 beta (IL‐1β), interleukin‐6 (IL‐6), interleukin‐8 (IL‐8), and tumor necrosis factor‐alpha (TNF‐α) [18]. More significantly, *P. gingivalis* degrades complement components, impairs neutrophil functionality, and inhibits phagocytosis, thereby exacerbating inflammatory damage and immune evasion. This cascade of effects leads to a perturbation in microbial homeostasis and accelerates the progression of periodontal inflammation [19]. *Fn*, a bridging microorganism, facilitates the translocation of key pathogens like *P. gingivalis* to periodontal infection sites, enhancing their pathogenicity [20]. In human gingival fibroblasts (hGFs) infected with *Fn*, NADPH oxidase isoforms NADPH oxidase 1 (NOX1) and NADPH oxidase 2 (NOX2) are activated, generating ROS [21]. Furthermore, what was formerly known as aggressive periodontitis is now classified as periodontitis with a rapid rate of progression (Grade C), which predominantly affects systemically healthy adolescents or young adults [22]. It is characterized by rapid periodontal attachment loss and severe alveolar bone destruction that is often disproportionate to the amount of dental plaque [23]. *Aa*, a facultative anaerobic Gram‐negative coccobacillus, is widely regarded as a key pathogen in this condition [24]. To establish infection, trigger host tissue damage, and ensure survival, *Aa* produces leukotoxin A (LtxA), which specifically targets the LFA‐1 receptor on neutrophils. This interaction leads to membrane disruption, calcium influx, and ultimately, neutrophil lysis [25]. Before cell death, neutrophils release large quantities of ROS and matrix metalloproteinases (MMPs), exacerbating local tissue destruction [26]. Beyond leukotoxin activity, *Aa* expresses cytolethal distending toxin (CDT), which induces DNA damage and cell cycle arrest in macrophages [27]. CDT is strongly associated with aggressive forms of periodontitis and contributes to alveolar bone degradation by upregulating receptor activator of nuclear factor kappa‐B ligand (RANKL), a key regulator of osteoclastogenesis [28]. Moreover, *Aa*‐derived LPS has been shown to elevate inducible nitric oxide synthase (iNOS) expression and stimulate nitric oxide (NO) production in osteoblast‐like cells. Excessive NO generation in response to microbial challenge may further accelerate bone loss [29].

Taken together, the interplay between ROS and periodontal pathogens encompasses immune defense mechanisms, pathogen evasion strategies, and resultant OS, all of which collectively facilitate the pathogenesis of periodontal disease.

### 2.2 Hyper‐Reactive PMNs

PMNs are the most common inflammatory cells in periodontal tissues, essential for maintaining periodontal homeostasis [30]. However, over‐activated PMNs produce excessive ROS, leading to tissue destruction in periodontitis [31]. The amount and activity of neutrophils during phagocytosis influence the extent of ROS production in the oral cavity [32]. NOX2, the most common NOX enzyme complex, is found in neutrophils and macrophages [33]. It mediates electron transfer from intracellular NADPH to the cell membrane, where it reacts with oxygen to form superoxide anions, a type of ROS, in a process called respiratory burst [34].

In periodontitis development, when early periodontal pathogens invade, a large number of PMNs are recruited and activated in the damaged area to clear the periodontal pathogens by releasing ROS [35]. However, with prolonged stimulation of chronic periodontal inflammation, PMNs reach a highly activated state, leading to excessive ROS production [36]. Carina et al. have already demonstrated that neutrophils in periodontitis patients are overreactive and produce higher levels of ROS in both spontaneous and stimulated situations compared to healthy individuals [3]. This induces OS, causing irreversible damage to the periodontal tissues [37].

### 2.3 Mitochondrial Dysfunction

Mitochondrial dysfunction is a significant contributor to periodontitis, driving excessive ROS accumulation, OS, and dysregulated mitochondrial dynamics [38]. Mitochondrial ROS (MtROS) are primarily produced by Complex I and Complex III of the electron transport chain (ETC) located on the inner mitochondrial membrane [39]. Complex I generate O2‐ in the mitochondrial matrix, where superoxide dismutase 2 (SOD2) converts it to H_2_O_2_ [40, 41]. Complex III generates O2‐ both in the matrix and intermembrane space (IMS), from where it can translocate into the cytoplasm [42, 43]. During oxidative phosphorylation, electrons from intermediate metabolites are transferred through the ETC to reduce oxygen to water [44]. However, approximately 1%–3% of these electrons can prematurely leak, particularly from Complexes I and III, resulting in the formation of superoxide anions even under resting conditions [45]. Under normal circumstances, endogenous antioxidant systems—including SOD2, catalase, and glutathione peroxidase—effectively neutralize ROS and maintain redox balance [46]. Under pathological conditions such as periodontitis, bacterial pathogens such as *P. gingivalis* and chronic inflammatory stimuli can aggravate mitochondrial dysfunction, enhancing electron leakage and overwhelming antioxidant defenses [47]. MtROS, when accumulated, not only serve as deleterious oxidants but also function as critical secondary messengers that activate redox‐sensitive signaling cascades such as the NF‐κB pathway, thereby promoting the transcriptional upregulation of pro‐inflammatory mediators including TNF‐α, IL‐1β, and IL‐6 [48]. Furthermore, the cytosolic release of mitochondrial DNA (mtDNA), acting as a damage‐associated molecular pattern (DAMP), initiates NLRP3 inflammasome activation and amplifies the secretion of inflammatory cytokines [49]. Excessive mtROS also facilitate the formation of neutrophil extracellular traps (NETs), which, in turn, release MMPs that degrade periodontal connective matrices. Additionally, sustained mitochondrial calcium overload driven by elevated ROS levels can induce the opening of the mitochondrial permeability transition pore (mPTP), ultimately triggering apoptosis. Furthermore, impaired mitophagy due to dysfunction of the PI3K/AKT pathway compromises mitochondrial quality control, thereby exacerbating OS [50]. Collectively, mtROS originating from electron leakage forge a pivotal mechanistic link between OS and the progressive destruction of periodontal tissues.

Abnormal mitochondrial function has been observed in gingival tissues and fibroblasts in chronic periodontitis, with *P. gingivalis* exacerbating mitochondrial damage [51]. Xu et al. [38] demonstrated that, *P. gingivalis* induces mitochondrial dysfunction through Drp1, resulting in mitochondrial fragmentation, elevated MtROS, and reduced ATP production. Additionally, mitophagy, which regulates inflammation, is disrupted by MtROS, intensifying inflammation in periodontitis. Activating mitochondrial autophagy may represent a promising therapeutic target [52]. Previous researches showed that mtROS production decreases in immune cells after periodontal treatment, correlating with improved endothelial function in patients with periodontitis [53].

### 2.4 Unhealthy Lifestyle Factors

Harmful lifestyle choices, such as consuming a diet high in fats and proteins, smoking, and excessive alcohol intake, significantly increase the risk of developing periodontitis. These behaviors contribute to elevated ROS levels in the oral environment, exacerbating inflammation in periodontal tissues and promoting alveolar bone resorption.

High‐fat and high‐protein diets have a detrimental effect on periodontal health, which is often associated with higher OS [54, 55]. High‐calorie intake results in an overabundance of electrons transferred to the ETC, sharply increasing superoxide anion production. When the respiratory chain remains chronically overloaded, redox imbalances occur throughout the body, including the oral cavity [56]. Peripheral blood neutrophils (PBN) from obese patients exhibit increased ROS production and cytokine release, contributing to heightened local and systemic inflammation [57]. Interestingly, both ROS production and inflammatory factor release are reduced after bariatric surgery [58].

Smoking is recognized as a risk factor in periodontal disease and other oral conditions [59]. Nicotine, a major component of cigarette smoke, directly increases the respiratory burst in neutrophils, impairing their function and triggering ROS production, which leads to OS‐related tissue damage [59, 60]. Simultaneously, when saliva is exposed to cigarette smoke, it triggers an increase in oxidant production, resulting in protein modifications in the carbonyl form and reduced enzyme activity. This weakens salivary defense mechanisms, potentially leading to various oral diseases [61]. Chronic alcohol consumption, on the other hand, can result in mitochondrial dysfunction, impairing cellular functions. ROS are generated during all stages of alcohol metabolism, including the processes catalyzed by alcohol dehydrogenase and mitochondrial enzymes, which oxidize alcohol into superoxide anions and hydroxyl radicals, respectively [62]. Chronic alcohol intake also lowers salivary amylase activity. Therefore, avoiding smoking and excessive alcohol consumption is crucial in reducing the risk of periodontitis and other oral diseases.

## 3 The Dual Role of ROS in Periodontal Tissue

### 3.1 The Physiological Effects of Appropriate ROS Levels

ROS play a crucial role in maintaining cellular metabolism and physiological processes. They are produced in various cellular locations, including the mitochondria, cytoplasm, and endoplasmic reticulum (ER) [63, 64, 65]. Under normal conditions, when cells are exposed to hydrogen peroxide, SOD1 relocates to the nucleus and binds to promoters to upregulate genes that control antioxidant defenses, ensuring a balance between ROS production and antioxidant systems [66]. Low ROS levels are crucial for sustaining intracellular redox homeostasis, which includes signal transduction, redox regulation, and defense mechanisms [67].

**Figure 1:**
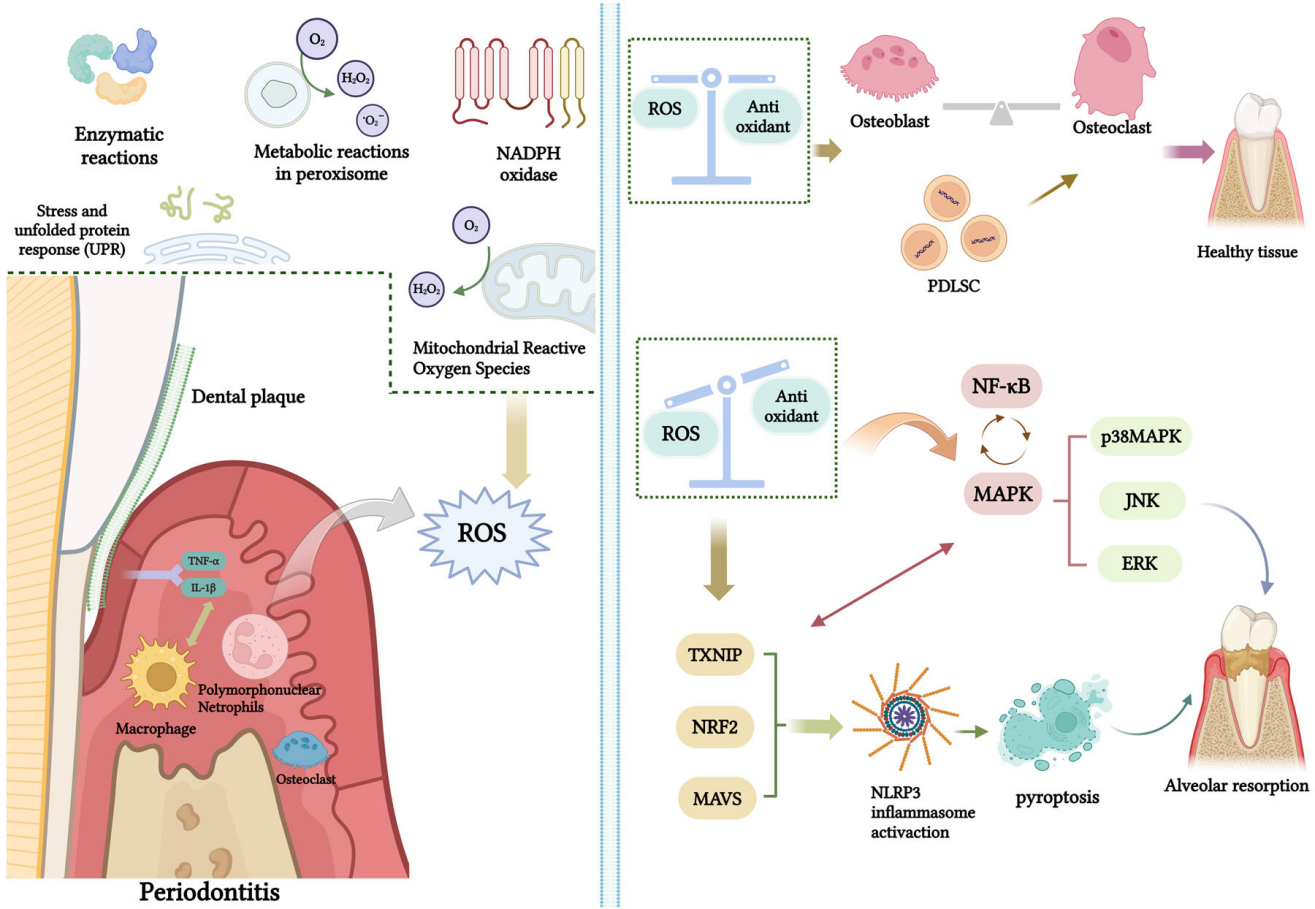
The dual role of ROS in periodontal tissue. In periodontal tissues, pathogen invasion induces host cells to release pro‐inflammatory factors like IL‐1β and TNF‐α, recruiting PMNs and macrophages to the inflammation site. These inflammatory cells eliminate pathogens by releasing ROS. (1) **Appropriate levels of ROS**: are crucial for maintaining the healthy physiological function of periodontal tissues, thereby creating a favorable environment for bone regeneration. (2) **Excessive levels of ROS**: elicit an OS response, contributing to osteoclastic bone resorption via modulation of diverse signaling pathways (created with BioRender.com/u03r009).

In periodontal tissues, when pathogens invade, host cells release pro‐inflammatory cytokines, such as interleukins and TNF‐α, which attract immune cells like PMNs and macrophages to the inflamed site [68]. These immune cells eradicate pathogens by releasing ROS [32] (Figure 1). Thus, maintaining appropriate ROS levels allows periodontal tissues to perform their normal defensive functions. Additionally, physiological levels of ROS are vital for preserving stem cell functions, which are essential for balancing stem cell differentiation and self‐renewal [69]. Periodontal ligament stem cells (PDLSCs) play an important role in periodontal regeneration, and maintaining their function is crucial for tissue repair [70]. Research by Qiu et al. [71] shows that appropriate ROS levels are important for promoting effective bone regeneration of PDLSCs within the periodontal environment.

### 3.2 The Pathogenic Effects of Excessive ROS Levels

Prolonged exposure to elevated ROS levels that exceed the body's antioxidant defenses leads to OS [72]. Excessive ROS disrupt cellular integrity, damaging proteins, mitochondria, and DNA, which result in tissue impairment and a variety of diseases [73]. Chronic inflammatory disorders, including periodontitis, rheumatoid arthritis, cardiovascular disease, and obesity, are linked to elevated levels of ROS [74, 75, 76, 77, 78, 79].

In periodontal tissues, immune cells often release excess ROS in response to pathogens, disrupting the equilibrium between ROS generation and antioxidant defenses, ultimately resulting in OS [80]. This oxidative imbalance is the primary cause of periodontal tissue destruction. Excessive ROS act as signaling molecules within cells, activating inflammatory pathways and inflammasomes, thereby increasing the release of pro‐inflammatory cytokines and worsening periodontal inflammation. Excessive ROS activate inflammatory pathways like NF‐κB and MAPK, promoting alveolar bone loss. ROS also trigger NLRP3 inflammasome activation by inducing the expression of OS‐related proteins like TXNIP, Nrf2, and MAVS, further contributing to tissue damage (Figure 1). Elevated intracellular ROS levels enhance the inflammatory response, with M1‐polarized macrophages further escalating the secretion of inflammatory cytokines and recruiting additional neutrophils to the inflamed site, thus accelerating the progression of periodontitis [81]. Thus, ROS are therefore critical in chronic inflammation, tissue matrix degradation, and bone remodeling.

## 4 Mechanisms of ROS in the Pathological Condition of Periodontal Tissues

**Figure 2:**
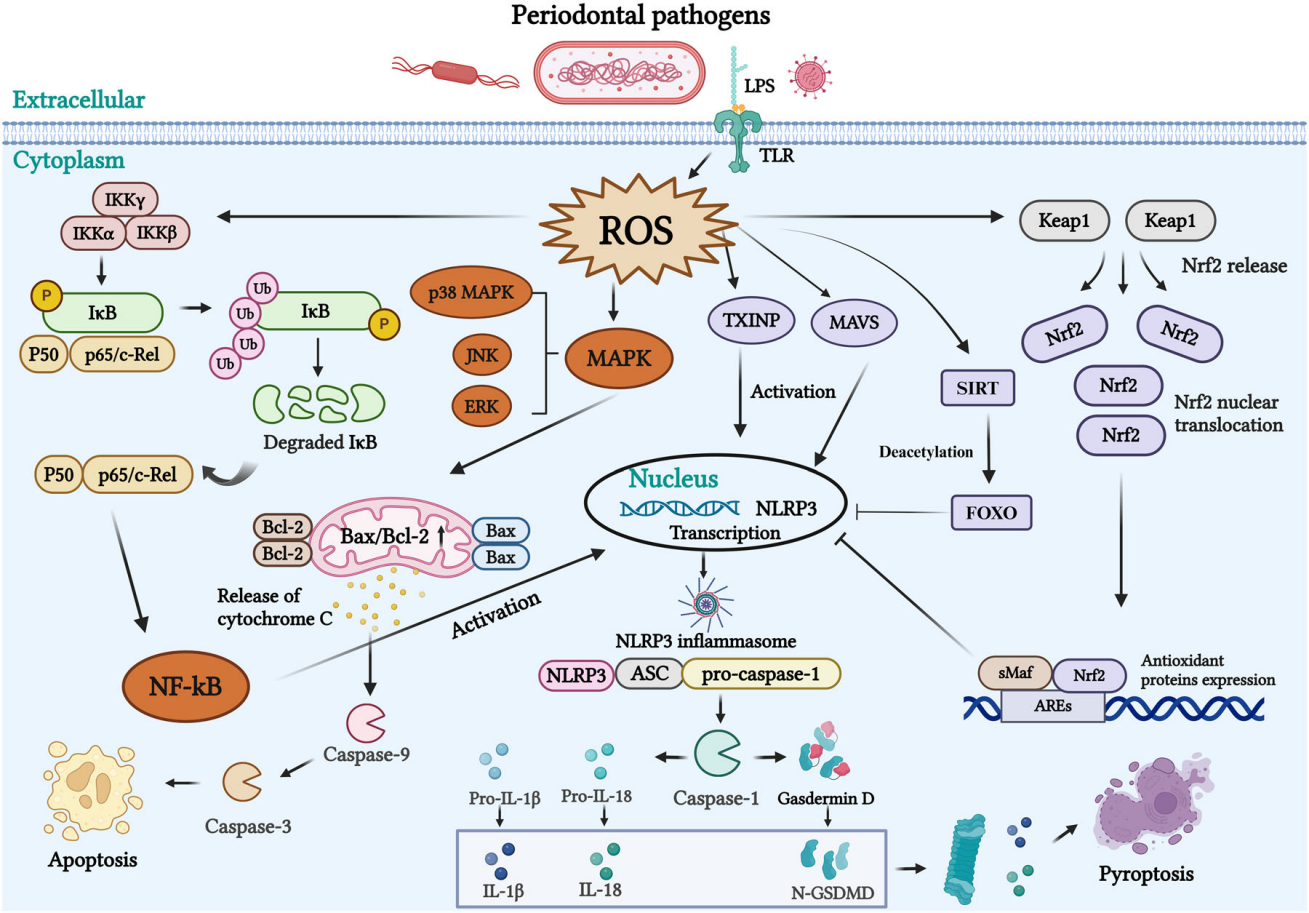
Specific ROS mechanisms in the pathological state of periodontal tissues. When exposed to external stimuli like *P. gingivalis* and LPS, intracellular ROS levels rise. These processes interact with each other, accelerating periodontitis and ultimately causing alveolar bone resorption. (1) **Excessive ROS modulate several signaling pathways. ROS and NF‐kB**: ROS and NF‐κB: The cytoplasmic heterodimer p65/p50 is activated by the IκB kinase complex, which weakens IκB's inhibitory effect on NF‐κB. Once free, NF‐κB translocates to the nucleus, promoting the expression of pro‐inflammatory genes, including those associated with NLRP3 inflammasome activation. **ROS and MAPK**: This signaling involves pathways such as p38 MAPK, JNK, and ERK. Bax/Bcl‐2 ratios are crucial in determining cell survival or apoptosis. Bax translocates to mitochondria, where it promotes cytochrome C release, which activates caspase‐9, resulting in the downstream activation of caspase‐3, ultimately inducing apoptosis. (2) **ROS also stimulate the NLRP3 inflammasome by activating oxidative stress‐related proteins. ROS and TXNIP**: High ROS levels reduce TRX activity, enhancing TXNIP's ability to activate NLRP3 inflammasome, increasing IL‐1β production. TRX dissociates from TXNIP in a ROS‐dependent manner, and activated TXNIP directly activates NLRP3 inflammasome, leading to increased production of mature IL‐1β. **ROS and MAVS**: MAVS recruits NLRP3 to mitochondria, enhancing its oligomerization and activation, which promotes caspase‐1 and IL‐1β production. (3) **Cells counteract oxidative stress by producing enzymes that neutralize ROS. ROS and Nrf2**: Nrf2 dissociates from Keap1 and moves to the nucleus, where it associates with AREs to regulate the expression of antioxidant genes. **ROS and SIRT/FOXO**: Enhanced SIRT1 activation alleviates oxidative damage by deacetylating FOXO (created with BioRender.com/b37w148).

An imbalance between ROS and antioxidant defenses initiates an OS response, contributing to periodontal tissue destruction [82]. A deeper understanding of these mechanisms is vital for developing comprehensive strategies for managing periodontal disease. The specific mechanisms of ROS in periodontitis are illustrated in Figure 2.

### 4.1 ROS and NF‐κB Signaling Pathway

NF‐κB functions as a crucial transcription factor activated by ROS and is essential for the regulation of OS and inflammatory processes [83]. In the classical NF‐κB activation pathway, cytoplasmic p65 and p50 heterodimers are typically inhibited by IκBα, keeping them inactive. Upon external stimulation, IκB kinase is activated, leading to IκBα degradation. This free NF‐κB translocates to the nucleus, where it promotes the expression of NLRP3 inflammasome‐associated genes [84].

Numerous external stimuli, such as bacterial infections and virulence factors, increase ROS production [85]. Both Gram‐negative anaerobes and microaerophiles can destabilize the redox equilibrium [86]. *P. gingivalis*, a primary bacterium involved in periodontitis, disturbs the balance between beneficial and harmful oral bacteria [87]. LPSs from *P. gingivalis* increase OS in periodontal ligament fibroblasts (PDLFs), contributing to periodontitis [88]. LPS also elevates intracellular ROS production, activating the NF‐κB p65 pathway, which enhances the release of inflammatory cytokines like TNF‐α and IL‐1β. Antioxidants such as *N*‐acetyl‐l‐cysteine (NAC) reduce *P. gingivalis*‐induced NF‐κB activation and cytokine expression by neutralizing ROS [89]. Moreover, recent research has highlighted the correlation between psychological stress and periodontitis. Some studies suggest that psychosocial stress may have a more significant impact on periodontal inflammation than pathogenic bacteria [90, 91]. Anxiety, depression, distress, and perceived stress affect a substantial percentage of periodontitis patients, with rates of 35.2%, 18.2%, 24.6%, and 43.9%, respectively [92]. Stress hormones promote the proliferation of Gram‐negative bacteria like *P. gingivalis*, fostering an environment conducive to periodontal tissue destruction [93]. Chronic psychological stress further causes microcirculatory disorders, leading to congestion and localized hemorrhaging in periodontal tissues. This transformation shifts the periodontal environment from normoxia to hypoxia [94]. Hypoxia, in combination with inflammation, activates hypoxia‐inducible factor‐1 (HIF‐1) [95].

ROS can both activate and inhibit NF‐κB signaling across various cell types, including macrophages, PMNs, human periodontal ligament cells (hPDLCs), hGFs, human gingival mesenchymal stem cells (hGMSCs), osteoblasts, and osteoclasts [96, 97, 98]. These effects are mediated through upstream pathways such as the Toll‐like receptor (TLR) and MAPK pathways [99]. ROS‐induced NF‐κB activation leads to the expression of pro‐inflammatory cytokines and chemokines, driving periodontal tissue destruction by triggering inflammation and osteoclast differentiation [100, 101]. ROS activation triggers the NF‐κB signaling pathway and enhances the release of IL‐6, IL‐1β, IL‐1, IL‐2, TNF‐α, prostaglandin E2 (PGE2), and lymphotoxin, etc [102]. These cytokines and chemokines are associated with bone resorption in periodontal tissues, with PGE2 being the most potent stimulator of periodontal bone resorption, IL‐1 and TNF‐α initiating bone resorption in periodontitis, IL‐1 being the most powerful inducer of bone demineralization, and IL‐6 stimulating bone resorption while simultaneously suppressing bone formation [103]. Nevertheless, ROS can also exert inhibitory effects on NF‐κB signaling under certain conditions. Excessive ROS accumulation may result in oxidative modifications of critical signaling elements, such as cysteine residues within the DNA‐binding domains of NF‐κB subunits (e.g., p50 and p65), thereby compromising their DNA‐binding capacity and diminishing transcriptional activation [104]. Furthermore, ROS can oxidize and inactivate components of the IκB kinase (IKK) complex, thereby preventing the phosphorylation and subsequent degradation of IκBα, ultimately obstructing NF‐κB nuclear translocation and activation [105]. In addition, ROS may induce the activation of antioxidant and cytoprotective pathways, notably the nuclear factor erythroid 2–related factor 2 (Nrf2) pathway, which antagonizes NF‐κB‐driven transcription by competing for transcriptional coactivators or by upregulating anti‐inflammatory genes such as Heme Oxygenase‐1 (HO‐1) and NAD(P)H Quinone Dehydrogenase 1 (NQO1) [106]. Elevated ROS levels attenuate NF‐κB signaling and suppress the expression of pro‐inflammatory cytokines, potentially serving as a compensatory mechanism to mitigate tissue injury under conditions of OS. Thus, ROS acts as a double‐edged sword in the regulation of NF‐κB signaling, with its effects being highly context‐dependent and influenced by factors such as ROS concentration, duration of exposure, and the intracellular redox environment.

### 4.2 ROS and MAPK Signaling Pathway

MAPK family is composed of serine‐threonine kinases that regulate numerous cellular processes, such as proliferation and apoptosis [107]. There are three key MAPK pathways: p38 MAPK, c‐Jun N‐terminal kinase (JNK), and extracellular signal‐regulated kinase (ERK), which are all crucial for transmitting extracellular signals to the nucleus [108]. MAPK pathways are highly sensitive to ROS and function as redox‐sensitive molecules that detect and transmit ROS signals [109]. These pathways are not only integral to normal physiological processes, such as cell differentiation and proliferation, but also play essential roles in pathological conditions like apoptosis, tissue injury, OS, and inflammation. Their primary mechanism involves extracellular stimuli activating specific upstream phosphorylation sites, triggering the regulation of apoptosis [110, 111]. The survival of a cell or its progression toward apoptosis is governed by the interplay between the anti‐apoptotic protein B‐cell lymphoma 2 (Bcl‐2) and the pro‐apoptotic protein Bcl‐2‐associated X protein (Bax) [112]. Bax translocates to the mitochondria, enhancing cytochrome C release, which then activates caspase‐9 and subsequently initiates the caspase‐3 cascade, leading to apoptosis [113, 114]. Li et al. [115] established a periodontitis model in beagle dogs through *P. gingivalis* infection and found that PBM therapy enhances OS by modulating the ROS/MAPK/mTOR signaling pathway. This approach regulates various inflammatory factors, boosts anti‐inflammatory and antioxidant capacities in saliva, improves bone density, and promotes implant osseointegration, thereby aiding in the prevention of periodontitis‐related infections [115].

#### 4.2.1 p38 MAPK Signaling Pathway

p38 MAPK, a key MAPK family member, is primarily involved in responses to OS, inflammation, and apoptosis [116]. Hypoxia has been demonstrated to induce cell death in human periodontal fibroblasts (PLFs), exacerbating periodontitis progression [117]. As mitochondria are a major source of ROS, superoxide production by the ETC increases under hypoxic conditions, with additional sources like NOX also generating oxidative signals in response to hypoxia, causing OS in periodontal tissues [118]. Studies have shown that both hypoxia and LPS‐Pg stimulation elevate NOX4‐dependent H₂O₂ release in PLFs [88]. Furthermore, oxygen deficiency enhances reductive carboxylation, leading to an increase in ROS production [119]. Hypoxia activates p38 MAPK, which translocates to the nucleus to regulate cell proliferation and apoptosis. Osteopontin inhibits the expression of pro‐apoptotic proteins, including Bax and caspase‐3, while simultaneously enhancing the expression of Bcl‐2. Inhibiting p38 MAPK with osteopontin could reduce OS and improve the antioxidant capacity of PLFs, preventing hypoxia‐induced cell death [120, 121].

#### 4.2.2 JNK Signaling Pathway

As the stress‐activated protein kinase, JNK plays a pivotal role in immune regulation [122]. JNK phosphorylation facilitates Bax relocation to the outer mitochondrial membrane, where Bax either functions as a pore‐forming protein or interacts with voltage‐gated anion channels (VDAC). The permeabilization of the mitochondrial membrane allows for the expansion of the outer membrane pore, participating in ROS‐induced apoptosis [123, 124]. Elevated glucose levels in patients with Type II diabetes mellitus (T2DM) lead to the formation of non‐enzymatic advanced glycation end products (AGEs) in an oxidative environment, which can accumulate in periodontal tissues, potentially resulting in their involvement in periodontitis [125]. TNF‐α and AGEs stimulate endogenous ROS production in PDLSCs. The continued ROS accumulation harms PDLSCs, leading to the activation of the JNK pathway, a decrease in mitochondrial membrane potential, the activation of caspase‐3, and an elevated Bax/Bcl‐2 ratio [126]. Recent studies have revealed that LPS‐preconditioned DFC‐sEVs demonstrate significant advantages in suppressing alveolar bone loss and promoting regeneration in dogs with experimental periodontitis. These effects may be attributed to the inhibition of ROS/JNK signaling, which reduces the RANKL/OPG ratio in PDLSCs, and the promotion of macrophage polarization toward the M2 phenotype via ROS/ERK signaling [127].

#### 4.2.3 ERK Signaling Pathway

ERK is activated by the accumulation of hydrogen peroxide (H_2_O_2_), a reactive oxygen metabolite produced in the cell. Activated ERK moves to the nucleus to perform various functions and plays a key role in cellular reactions to OS [128]. ROS are central in activating the NLRP3 inflammasome [129]. ERK‐specific inhibitors suppress NLRP3‐mediated caspase‐1 activation and IL‐1β secretion while also inhibiting NF‐κB activation, indicating that ERK is upstream of both NF‐κB and the NLRP3 inflammasome [130]. The ERK pathway plays an essential role in apoptosis. Phosphorylated ERK1/2 triggers apoptosis in NRK‐52E cells by decreasing Bcl‐2 expression, increasing caspase‐3 and Bax levels, and elevating ROS [131, 132]. Mesenchymal stem cells (MSCs) are the main source of stem cells involved in periodontal bone regeneration. Ren et al. [133] showed that MSCs stimulate the extracellular signal‐regulated kinase (ERK)/AMP‐activated protein kinase (AMPK) pathway, enhancing the osteogenic potential of bone marrow MSCs [133]. Therefore, the ERK/AMPK pathway holds significant research potential for treating periodontitis.

### 4.3 ROS and NLRP3 Inflammasome

The NLRP3 inflammasome is widely distributed in tissues and organs throughout the body. It is the most prominent inflammasome in the NLR family and is involved in innate immune responses to infections, inflammation, and chronic diseases [134]. The NLRP3 inflammasome consists of three components: NLRP3, apoptosis‐associated speck‐like protein (ASC), which contains the caspase‐1 recruitment domain, and pro‐caspase‐1 [135]. NLRP3 activation occurs in two steps [1]: the initiation step, where pathogen‐associated molecular patterns (PAMPs) such as LPS and cytokines such as TNF‐α, activate NF‐κB, leading to the upregulation of genes associated with the NLRP3 inflammasome [136], and [2] the activation step, initiated by PAMPs or DAMPs like ROS, bacterial infections, ion efflux, or mitochondrial damage, which leads to NLRP3 assembly and caspase‐1 activation [137]. LPS from *P. gingivalis* is a significant PAMP that binds to TLR4, facilitating inflammatory mediator release [138]. ROS generation in response to LPS is a crucial signal in the inflammatory process, and ROS also trigger NLRP3 inflammasome activation, contributing to periodontitis progression [139].

Excessive NLRP3 inflammasome activation is associated to numerous inflammatory conditions, including diabetes, Alzheimer's disease, gout, atherosclerosis, and periodontitis [140]. The identification of the NLRP3 inflammasome offers a key regulatory pathway for investigating periodontitis. Activated NLRP3 converts pro‐inflammatory IL‐1β into its mature, active form via caspase‐1 [136]. IL‐1β is a major cytokine associated with the progression of periodontitis, as it enhances osteoclast formation and promotes bone resorption [96]. The effects of the NLRP3 inflammasome on osteoclastogenesis vary depending on context. In the presence of infections like LPS, NLRP3 promotes osteoclast formation, leading to tissue destruction. However, in non‐infectious conditions, NLRP3 inhibits osteoclastogenesis by inducing apoptosis in osteoclast precursors [141].

As a form of programmed cell death driven by inflammation, pyroptosis is also involved in periodontal tissue degradation [142]. Unlike apoptosis and autophagy, pyroptosis is primarily mediated by caspase and gasdermin (GSDM) proteins [143]. NLRP3 inflammasome and caspase‐1 activate pyroptosis by converting pro‐caspase‐1 into its active form, which cleaves substrates like pro‐IL‐1β and pro‐IL‐18 into their active forms [144]. Caspase‐1 also cleaves gasdermin D (GSDMD), leading to the formation of membrane pores that facilitate the release of IL‐1β and IL‐18, thereby initiating a robust inflammatory response [145]. Additionally, GSDMD, the key effector of pyroptosis, forming pores in the cell membrane and allowing the release of inflammatory cytokines like IL‐1β and IL‐18, thereby triggering a robust inflammatory response [146]. In gingival tissues, the expression levels of NLRP3, Caspase‐1, and IL‐1β were significantly higher in patients with periodontitis than in healthy individuals [147]. A multitude of studies has demonstrated the pivotal role of the ROS/NLRP3 inflammasome in the development and progression of periodontitis.

LPS, the primary PAMP in periodontitis, binds to TLR4 and activates a signaling cascade that promotes the release of inflammatory factors and ROS [138]. ROS production in response to LPS also activate the NLRP3 inflammasome by inducing OS‐related proteins like TXNIP and MAVS, which further drive periodontitis progression [139].

#### 4.3.1 Thioredoxin‐Interacting Protein (TXNIP)

TXNIP, part of the thioredoxin (TRX) system, is a crucial regulator of OS, involved in cellular processes like proliferation, differentiation, and pyroptosis [148]. In its inactive state, TXNIP is bound to TRX in the cytoplasm and ER, where it forms disulfide bonds, inhibiting TRX function [149, 150]. When ROS levels rise, TRX activity decreases, and TXNIP becomes active, promoting NLRP3 inflammasome activation and increasing IL‐1β production [151, 152]. Studies have shown that PPARγ activation can reduce ROS levels and inhibit TXNIP/NLRP3 signaling, decreasing pyroptosis and improving organ function during sepsis [153]. Compounds such as cilantro protein have been found to inhibit TXNIP‐NLRP3 interaction, reducing ROS levels and cell pyroptosis, offering relief from NLRP3‐related conditions like gouty arthritis [154]. TXNIP is a vital link between ROS and NLRP3‐mediated pyroptosis [155].

Recent studies have highlighted the role of the ROS/TXNIP/NLRP3 pathway in periodontitis development. Research by Lian et al. [156] demonstrated that *P. gingivalis* lipopolysaccharide (Pg‐LPS) induces an inflammatory response in mouse PDLFs via the ROS/TXNIP/NLRP3 pathway, leading to chronic periodontitis in mice [156]. Additionally, Zhu et al. [157] found that obstructive sleep apnea‐hypopnea syndrome (OSAHS) increases NLRP3 and caspase‐1 activation in periodontal tissues, making them more vulnerable to inflammatory lesions through the ROS/TXNIP/NLRP3 pathway [157].

#### 4.3.2 Mitochondrial Antiviral Signaling Protein (MAVS)

MAVS, a mitochondrial membrane protein, is crucial for the production of Type 1 interferons and mediates NLRP3‐mitochondrial interactions [158]. Mitochondria are a significant source of ROS necessary for NLRP3 activation, and their role in pyroptosis underlines the critical role of mitochondria in the innate immune response [159]. MAVS detects ROS‐driven inflammation, and its aggregation amplifies ROS production [160]. Previous studies reported that ROS are essential for initiating NLRP3 activation but not necessary for sustaining it [161]. MAVS functions within the NLRP3 inflammasome by recruiting TLRs and RIG‐I, mediating NF‐κB and Type I interferon signaling [155, 158]. MAVS also promotes NLRP3 oligomerization, leading to the secretion of caspase‐1 activation and IL‐1β [162].

Research has demonstrated that Suyin Detoxification Granules (SDG) protect renal tubular epithelial cells from apoptosis by regulating the MAVS/NLRP3 pathway [163]. NLRP3 inflammasome within these cells produces MtROS, exacerbating damage following hypoxia through interactions with MAVS [164]. Nevertheless, there is currently no literature reporting on the function of the ROS‐MAVS‐NLRP3 axis in periodontitis. Given the significance role of ROS and NLRP3 in the development of periodontitis, the ROS‐MAVS‐NLRP3 axis may become a potential avenue for future research as one of the pathogenesis and therapeutic targets of periodontitis.

ROS precipitate the degradation of periodontal tissues. To counteract OS, cells produce protective enzymes that neutralize ROS. Key regulatory pathways governing the production of these protective enzymes include NRF2, silent information regulator T (SIRT), and FOXO [165].

#### 4.3.3 Nuclear Factor Erythroid 2‐Related Factor 2 (Nrf2)

Nrf2 functions as a key sensor for OS and plays a crucial role as a transcription factor in suppressing ROS‐induced inflammation [166]. It regulates the expression of various antioxidant enzymes, such as heme oxygenase‐1 (HO‐1), glutathione‐S‐transferase (GST), cystathionine ligase, and NADPH quinone oxidoreductase 1 (NQO1), thereby offering protection against OS [167]. In a typical physiological state, Nrf2 is predominantly found in the cytoplasm, where it is tethered to its negative regulator, Kelch‐like ECH‐associated protein 1 (Keap1). This binding leads to the ubiquitination and degradation of Nrf2 [168]. However, during OS, Nrf2 detaches from Keap1 and moves into the nucleus. There, it interacts with AREs, initiating the transcription of genes responsible for antioxidant defense [169, 170].

Recent studies suggest that activating Nrf2 can prevent excessive ROS production and inhibit the activation of the NLRP3 inflammasome, positioning Nrf2 as a potential target for the treatment of inflammatory diseases [171, 172]. Consequently, therapies that enhance Nrf2 signaling may provide a promising strategy to prevent inflammation‐related diseases. Research by Chen et al. [173] demonstrated significant expression of Nrf2, HO‐1, NLRP3, and ASC in the synovial tissues of patients with osteoarthritis. Their findings indicate that NLRP3 inflammasome activation is driven by ROS following LPS stimulation, with the anti‐inflammatory effects of the Nrf2/HO‐1 pathway explaining the variations in ROS levels [173].

It has been established that there is a close association between pyroptosis and periodontitis [174]. For instance, a recent study revealed that ED‐71, a new active vitamin D analog, can protect hGFs from LPS‐induced NLRP3 inflammasome‐mediated cell death via the Nrf2/HO‐1 signaling pathway [139]. Similarly, it was discovered for the first time that silymarin (SB), a natural polyphenolic flavonoid, reduces ROS levels in LPS‐induced human periodontal stem cells (hPDLCs) by downregulating NF‐κB and NLRP3, while upregulating Nrf2 expression. This demonstrates both anti‐inflammatory and antioxidant benefits, suggesting that SB may hold significant potential for clinical use in treating periodontitis [175].

#### 4.3.4 Forkhead Box O (FOXO)

FOXO1 is a ubiquitously expressed and evolutionarily conserved transcription factor, which includes FOXO1, FOXO3, FOXO4, and FOXO6 [176]. FOXO plays a pivotal role in orchestrating numerous vital biological processes, including cellular survival, differentiation, ROS attenuation, apoptosis, cellular proliferation, senescence, and the maintenance of stem cell homeostasis [177]. FOXO proteins are crucial in the cellular defense against OS by preventing ROS formation, sequestering reactive species, repairing damaged molecules, and inducing protective signaling pathways [178]. Pathogens like *P. gingivalis* and LPS trigger FOXO1 activation in neutrophils through TLR2 and TLR4, a process involving FOXO1 deacetylation and ROS generation [179]. The signaling pathways of TLR2 and TLR4 facilitate FOXO1 nuclear translocation, contingent upon ROS formation [180]. ROS, induced by *P. gingivalis*, augments FOXO nuclear translocation and activity via the JNK signaling cascade [181]. Activated FOXO mitigates OS by upregulating enzymes that decompose ROS. FOXO1 and FOXO6 alleviate OS by transcribing superoxide dismutase, thereby catalyzing the conversion of O_2_− to H_2_O_2_ [182]. As a result, FOXO1 safeguards PDLSCs against oxidative damage while also promoting their osteogenic potential in inflammatory conditions [183]. Though there are currently no direct studies linking FOXO to the NLRP3 inflammasome in periodontitis, research has shown that SIRT1 activation by SRT1720 promotes FOXO1 deacetylation, reduces ROS overproduction, and inhibits NLRP3 inflammasome activation in models of subarachnoid hemorrhage (SAH) [184]. This suggests a potential new pathway for periodontitis treatment.

#### 4.3.5 Silent Information Regulator T

SIRT refers to a group of Class III protein deacetylases that rely on NAD+ to function and regulate various physiological processes [185]. SIRT1, the most extensively studied member of the SIRT family, is a key modulator of redox balance and plays a vital role in cellular survival, apoptosis, and inflammation [184]. A study by G‐J et al. demonstrated for the first time that SIRT1 activation and overexpression provide significant protection against nicotine‐ and LPS‐induced cytotoxicity, reducing ROS production and pro‐inflammatory cytokine secretion via the PI3K, PKC, MAPK, and NF‐κB signaling pathways. This suggests that SIRT1 activation could help alleviate inflammation in periodontal diseases [186]. Additionally, SIRT1 protects MSCs from damage by inhibiting IL‐1β release through the NLRP3 inflammasome [187]. SIRT1 regulates FOXO1 by enhancing its ability to bind to DNA, which in turn promotes the expression of specific target genes. This modulation plays a key role in mitigating OS across a range of disease conditions [188]. In conclusion, SIRT1 is a promising therapeutic target for treating periodontitis by mitigating OS, reducing inflammatory responses, and restoring mitochondrial function.

## 5 Emerging Strategies for the Treatment of Periodontitis

Contemporary clinical approaches for managing periodontitis predominantly encompass mechanical debridement (including supragingival scaling, subgingival scaling, and root planing), adjunctive modalities (such as systemic antibiotic administration and localized antimicrobial therapies), and surgical interventions (such as flap surgery and guided bone regeneration [GBR] techniques). Despite their demonstrable efficacy in mitigating the progression of periodontitis, these interventions remain constrained by notable challenges and inherent limitations. Although mechanical debridement is universally acknowledged as the foundation of periodontal therapy and demonstrates efficacy in subgingival plaque biofilm removal, it frequently proves insufficient in completely eradicating bacterial biofilms within anatomically intricate sites, such as deep periodontal pockets, furcation defects, and root concavities [189]. Residual plaque biofilms serve as a primary source of persistent inflammation. Furthermore, while the administration of systemic antibiotics effectively reduces the burden of periodontal pathogens, the widespread use of these agents has precipitated the emergence of antibiotic resistance, now recognized as a global public health crisis [190]. Local antimicrobial therapies, such as the placement of metronidazole gels or minocycline hydrochloride ointment into periodontal pockets, have demonstrated significant efficacy in reducing localized pathogenic bacterial loads and mitigating inflammation. However, their limitations remain evident, primarily manifesting as short‐lived therapeutic effects, restricted areas of action, and reliance on the outcomes of concurrent mechanical debridement [191]. In summary, conventional treatment modalities exhibit notable limitations in eradicating pathogenic microorganisms, preventing disease recurrence, modulating the host inflammatory response, and promoting periodontal tissue regeneration. Furthermore, traditional therapies are often ineffective in addressing OS and inflammatory burden, which play pivotal roles in the pathogenesis and progression of periodontitis. Consequently, there is an urgent need to develop novel adjunctive therapeutic strategies to overcome the shortcomings of current approaches.

In recent years, as the understanding of the pathophysiological mechanisms underlying periodontitis has advanced, researchers have increasingly focused on innovative therapies aimed at enhancing the efficacy of conventional treatments. Antioxidants, PDT, and nanotechnology, recognized as highly promising therapeutic strategies, have emerged as key areas of investigation in the field of periodontal treatment. These emerging therapies not only aim to address the limitations of traditional approaches but also emphasize the modulation of host immune responses, alleviation of OS, and promotion of periodontal tissue regeneration, thereby offering novel insights and potential avenues for the treatment of periodontitis.

### 5.1 Antioxidants

Antioxidants are substances capable of significantly inhibiting or delaying oxidative reactions in substrates, even at low concentrations, thereby effectively mitigating OS [192]. In the clinical management of periodontitis, antioxidants can be categorized into endogenous and exogenous types. Endogenous antioxidants, such as superoxide dismutase (SOD), catalase (CAT), and glutathione peroxidase (GPx), play a pivotal role in counteracting OS within the body. However, their production is largely dependent on endogenous synthesis or exogenous supplementation [193, 194]. In contrast, exogenous antioxidants, such as vitamin C, melatonin, coenzyme Q10 (CoQ10), and polyphenolic compounds (including green tea catechins, resveratrol [RV], and curcumin), are typically administered via oral supplementation or topical application. These agents have been shown to effectively alleviate periodontal inflammation, promote periodontal tissue repair, and enhance therapeutic outcomes [37]. Given the significant therapeutic efficacy of exogenous antioxidants in the clinical management of periodontitis, we will next provide a concise overview of recent advancements in their clinical applications within this domain.

#### 5.1.1 Vitamin C

Vitamin C, a crucial water‐soluble vitamin, exhibits potent antioxidant and immunomodulatory properties. It is regarded as an essential dietary antioxidant for maintaining periodontal health [195]. Vitamin C supplementation mitigates OS by reducing the production of pro‐inflammatory cytokines in infected periodontal tissues. Additionally, it modulates NF‐κB‐DNA binding activity by inhibiting NF‐κB activation, thereby attenuating OS and inflammation‐induced periodontal tissue degradation [196]. Li et al. [197], through an analysis of data from the 2009–2014 National Health and Nutrition Examination Survey (NHANES) in the United States, investigated the relationship between dietary vitamin C intake and periodontitis. The study revealed that the risk of periodontitis was minimized at a dietary vitamin C intake of 158.49 mg, while both insufficient and excessive intake were associated with an increased risk of developing the disease [197]. Moreover, vitamin C supplementation has been shown to facilitate postoperative healing in patients undergoing GBR or Bio‐Oss collagen grafting as part of dental implant procedures [198].

#### 5.1.2 Melatonin

Melatonin exhibits a wide range of biological functions, including circadian rhythm regulation, antioxidant activity, and anti‐inflammatory properties [199]. As a potent antioxidant, melatonin exerts its effects by inhibiting the activity of nuclear transcription factors such as NF‐κB and MAPK, scavenging various free radicals, and modulating anti‐inflammatory mediators, positioning it as a promising biomarker and therapeutic agent [200]. Studies suggest that melatonin may alleviate periodontal tissue damage by suppressing the activation of the NLRP3 inflammasome, reducing cytokine expression, and preventing pyroptosis [201]. Recent clinical studies have confirmed that, in patients with Type 2 diabetes and periodontitis, supplementation with melatonin (6 mg/day for 30 days) significantly reduced periodontal inflammation and probing depth (PD), when compared to full‐mouth scaling and root planing (fmSRP) alone. Furthermore, evaluations based on clinical and biochemical markers, such as RANKL, OPG, MMP‐8 in gingival crevicular fluid, and serum IL‐1β levels, revealed no local or systemic adverse effects associated with melatonin supplementation [202]. Additionally, studies indicate that combining melatonin with an appropriate dosage of vitamin C may yield beneficial outcomes in the treatment of periodontitis, without causing detrimental effects on enamel due to its acidity [203].

#### 5.1.3 Coenzyme Q10 (CoQ10)

CoQ10 exhibits significant antioxidant activity by scavenging free radicals and enhancing the activity of key antioxidant enzymes, such as SOD, GPx, and CAT [204]. Studies suggest that CoQ10 may play a crucial role in the pathogenesis of periodontitis, as approximately 80% of periodontitis patients exhibit a deficiency of CoQ10 in gingival biopsies [205]. Yoneda et al. [206] were the first to demonstrate that CoQ10 can inhibit the phosphorylation of NF‐κB, thereby preventing its transcription factors from translocating into the nucleus and reducing the activation of the NLRP3 inflammasome, consequently diminishing the expression of inflammation‐related genes (such as IL‐1β and caspase‐1) in periodontal tissues [206]. Recent studies have further demonstrated that, compared to conventional periodontal therapy alone, CoQ10 supplementation (such as Perio Q™) significantly improves clinical parameters in patients with periodontitis, including plaque index (PI), gingival index (GI), gingival bleeding index, probing pocket depth, and attachment loss, with notable improvements observed at both 1 and 3 months post‐treatment [207]. Moreover, studies have indicated that both oral supplementation and local application of CoQ10 significantly ameliorate the symptoms of periodontitis, thereby supporting its role as an effective adjunctive therapy in the management of periodontal disease [208].

#### 5.1.4 Polyphenolic Compounds

Polyphenolic compounds are a class of natural organic substances characterized by multiple phenolic hydroxyl (OH) groups, primarily derived from plants [209]. Among them, epigallocatechin gallate (EGCG), RV, and curcumin have gained significant attention in recent clinical research due to their accessibility. These polyphenolic compounds exhibit remarkable antioxidant, anti‐inflammatory, antimicrobial, and immune‐modulatory effects, thereby garnering widespread interest as adjunctive therapies for periodontitis [210].

With the rising awareness of health, green tea has become one of the most widely consumed beverages globally due to its potential health benefits. Green tea is made from tea leaves that undergo minimal oxidation to preserve their natural polyphenolic content. It is rich in various polyphenolic compounds, particularly EGCG [211]. Recent studies have demonstrated that EGCG, the primary bioactive component of green tea, not only exerts significant effects in improving cardiovascular health and reducing blood lipids but also plays a crucial role in protecting the alveolar bone from excessive resorption through the modulation of various biological mechanisms [212]. High‐dose EGCG directly eliminates bacteria by disrupting their structural integrity, while low‐dose EGCG inhibits the activity of key virulence factors, reducing biofilm formation, and suppressing bacterial adhesion and aggregation, thereby diminishing their invasive impact on periodontal tissues [212]. Qin et al. found that EGCG (200 mg/kg) effectively reduced alveolar bone loss in a periodontal inflammation model by modulating the Nrf2/HO‐1/NLRP3/NF‐κB p65 signaling pathway, thereby inhibiting OS and inflammatory responses [213]. Additionally, Wang et al. [214] employed a novel dental scaler tip for delivering purified EGCG aqueous solution as a coolant in conjunction with scaling and root planing (SRP). The results revealed that, compared to SRP alone, the combined treatment significantly improved PD [214]. Furthermore, gingival patches (GP‐EGC) containing EGCG, which adhered to the mucosal membrane, demonstrated potential as an adjunctive therapy for periodontal disease by modulating the expression of IL‐6 and IL‐10 [215].

In recent years, the potential application of RV as an adjunctive therapy for periodontitis has garnered significant attention. Its mechanisms of action primarily encompass the regulation of OS, mitigation of inflammatory responses, and promotion of osteogenic differentiation. Studies have demonstrated that revealed that RV suppresses inflammation by downregulating the expression of TLR4, TNF‐α, and NF‐κB while activating ERK/Wnt signaling crosstalk, thereby enhancing the proliferation and osteogenic differentiation potential of GMSCs and significantly improving their immunomodulatory capabilities [216]. Zhang et al. [217] further evaluated the clinical efficacy of oral RV in patients with periodontitis. An 8‐week trial demonstrated that RV effectively reduced systemic and local inflammatory markers while significantly suppressing systemic endotoxin levels. The study also identified 500 mg/day as the optimal therapeutic dose of RV for periodontitis patients, providing crucial guidance for its application in clinical practice [217].

Curcumin, an antioxidant with diverse biological activities, is extracted from the roots of the turmeric plant [218]. Research has revealed that curcumin significantly inhibits osteoclast differentiation by suppressing the RANKL/RANK and NF‐κB signaling pathways while activating the Wnt/β‐catenin pathway, thereby effectively alleviating alveolar bone resorption [219]. Furthermore, clinical studies have demonstrated that curcumin exhibits comparable efficacy to the widely used antimicrobial agent chlorhexidine in reducing periodontal PD, improving clinical attachment loss (CAL), and decreasing GI and PI, providing robust support for its role as an adjunctive therapy in periodontal disease management [220].

#### 5.1.5 The Challenges and Limitations of Applying Antioxidants in Clinical Practice

As adjunctive therapies for non‐surgical periodontal treatment, antioxidants have demonstrated significant clinical potential but face numerous challenges and limitations in practical application. Firstly, many antioxidants exhibit low bioavailability due to their limited solubility, chemical instability, and rapid metabolism, which hinders their systemic or local efficacy. Moreover, individual variations in genetic background, immune status, and environmental factors (such as smoking, diet, and oral hygiene habits) can substantially influence the pharmacokinetics and pharmacodynamics of antioxidants. Secondly, the role of antioxidants within the complex periodontal microenvironment remains incompletely understood. The OS and inflammatory responses within periodontal tissues exhibit considerable heterogeneity, making it challenging for a single antioxidant to comprehensively address multiple sources of OS and inflammatory mediators. Furthermore, current research provides limited clinical data on the long‐term safety and potential side effects of antioxidants, complicating their clinical translation. Future advancements should prioritize optimizing drug delivery systems and exploring multifunctional synergistic therapies. The application of nanotechnology, including nanoparticles, liposomes, and hydrogels, holds promise for significantly enhancing the stability, targeting capability, and local concentration of antioxidants. Additionally, the investigation of combination therapies offers considerable potential, such as integrating antioxidants with antimicrobial agents, anti‐inflammatory drugs, osteogenic factors, or PDT to achieve multi‐target synergistic effects. These approaches aim to comprehensively inhibit pathological periodontal processes while promoting tissue regeneration. Furthermore, high‐quality, long‐term follow‐up randomized controlled clinical trials are urgently needed to validate the efficacy, safety, and feasibility of antioxidants for sustained application in periodontal therapy, thereby facilitating their widespread adoption in clinical practice.

### 5.2 Photodynamic Therapy

PDT, an emerging non‐surgical treatment modality for periodontitis, operates on the synergistic interaction of photosensitizers, specific wavelength light sources, and oxygen [221]. In recent years, PDT has been extensively utilized as an adjunct to traditional mechanical therapy to enhance therapeutic outcomes. During its application, photosensitizers, such as methylene blue or toluidine blue, are directly applied to periodontal pockets where they bind to pathogens and their biofilms. These photosensitizers are subsequently activated by light sources within a specific wavelength range (typically 630–650 nm) [222]. Upon activation, the photosensitizers transfer energy to produce singlet oxygen and other ROS, such as singlet oxygen (¹O₂) and hydroxyl radicals (·OH). These reactive molecules exert selective antimicrobial effects by effectively eliminating pathogenic microorganisms while also modulating inflammatory responses [223].

#### 5.2.1 The Advantages of PDT Compared With Conventional Therapy

PDT demonstrates significant advantages in selective antimicrobial activity, non‐invasive procedures, and anti‐inflammatory effects. Its unique photosensitizers exhibit a high specificity for binding to pathogenic microorganisms, effectively safeguarding host tissues from oxidative damage induced by ROS [224]. Since PDT relies on photochemical reactions rather than antibiotics for microbial eradication, it substantially mitigates the risk of developing antibiotic resistance [225]. By generating ROS, PDT effectively disrupts bacterial cell membranes and internal structures, showing potent bactericidal effects, particularly against anaerobic bacteria and Gram‐negative pathogens such as *Aa, P. gingivalis, and Prevotella intermedia (P. intermedia)*. These microorganisms are closely associated with the pathogenesis of periodontitis [226]. As a non‐invasive and painless therapeutic approach, PDT significantly alleviates patients’ treatment‐related fear and anxiety, particularly when addressing deep periodontal pockets or sensitive areas [227]. For individuals with systemic conditions such as diabetes, hypertension, or immunosuppression, PDT offers the advantage of minimizing complications associated with conventional therapies, including wound infections, bleeding, and poor healing outcomes [228]. Moreover, PDT demonstrates remarkable efficacy in inflammation control by suppressing the expression of pro‐inflammatory cytokines, such as IL‐1β and TNF‐α, thereby promoting periodontal tissue healing [229]. Studies have revealed that toluidine blue‐mediated photodynamic therapy (TB‐PDT) effectively mitigates inflammation by inhibiting NF‐κB signaling pathway activation, reducing the expression of RANKL and OPG, and modulating the OPG/RANKL ratio [230]. Additionally, research conducted by Jiang et al. [231] highlighted that methylene blue‐mediated photodynamic therapy (MB‐PDT) induces macrophage apoptosis via ROS, consequently reducing alveolar bone resorption [231]. Clinical trials further substantiate PDT's potential, demonstrating its efficacy in improving PD and AL, reducing inflammatory cytokine levels, and promoting periodontal tissue regeneration [232]. Nevertheless, the limitations of PDT warrant attention. These include the suboptimal penetration capacity of individual photosensitizers in tissues and biofilms, as well as their limited bioavailability, which collectively hinder their efficacy in accessing deeper periodontal pockets and may subsequently compromise therapeutic outcomes. Additionally, the therapeutic effectiveness of PDT can be influenced by various parameters, including the type and concentration of the photosensitizer, the wavelength and intensity of the light source, the duration of irradiation, oxygen availability, and interindividual variability among patients [233].

#### 5.2.2 The Combined Application of PDT and Antioxidants

In recent years, the combined application of antioxidant therapy and PDT has garnered significant attention. This combinatorial approach leverages antioxidants to modulate OS and inflammatory responses, not only alleviating periodontal inflammation but also augmenting the therapeutic efficacy of PDT. Curcumin, a natural photosensitizer, has been extensively studied for its integration into PDT for periodontal therapy. Its combination with PDT facilitates effective access to areas traditionally challenging to treat, markedly enhancing clinical outcomes [234]. Studies have demonstrated that curcumin‐mediated PDT effectively inhibits the growth of periodontal pathogens, such as Streptococcus mutans, with results significantly surpassing those achieved by curcumin alone [235]. Clinically, the combination of curcumin and PDT as an adjunct to SRP has shown remarkable antibacterial effects, particularly against periodontal pathogens such as *Aa, P. gingivalis*, and *P. intermedia*. Moreover, this synergistic therapy exhibits outstanding efficacy in improving clinical parameters, including significant reductions in bleeding on probing (BOP), PD, and CAL [236]. Recent studies have further substantiated the potential of integrating curcumin‐mediated PDT with 660 nm laser irradiation and increasing irradiation frequency. This approach fosters an optimal biological environment conducive to periodontal tissue regeneration [237]. Collectively, these findings underscore the potential value of curcumin‐mediated PDT as an adjunctive therapeutic strategy in periodontal disease management.

#### 5.2.3 The Application of Nanotechnology in PDT

With the rapid progress of nanotechnology, various functional nanomaterials have demonstrated tremendous potential in drug delivery and antimicrobial therapy for the treatment of periodontitis [238]. In recent years, the combination of PDT and gas therapy has led to the development of multifunctional nanomaterials, which have gained increasing attention in the field of dentistry [239]. For instance, Zeng et al. [240] developed a self‐propelled nanovesicle (PCL‐PLG@CHX) that generates NO through the reaction of guanidine groups with ROS. This system effectively eradicates bacterial biofilms within periodontal pockets, reduces inflammatory cell infiltration, and promotes angiogenesis and collagen repair [240]. For diabetic periodontitis, Wang et al. [241] designed a multi‐enzyme synergistic nanoplatform (MSN‐Au@CO) based on carbon monoxide (CO) gas therapy. This platform leverages the multi‐enzyme catalytic activity of gold nanoparticles (Au NPs) to lower local glucose concentrations and eradicate bacteria while utilizing a manganese‐based complex (MnCO) to release CO in response to hydrogen peroxide (H₂O₂) and hydroxyl radicals (·OH). The released CO modulates the Nrf2 and NF‐κB signaling pathways, demonstrating significant anti‐inflammatory, antibacterial, and bone loss‐preventing effects, with promising applications in diabetic periodontitis models [241]. The introduction of nanotechnology has opened new avenues for PDT research. Nanocarrier systems significantly enhance the distribution and penetration of photosensitizers within periodontal pockets, thereby increasing drug concentration at sites of deep infection. For example, Shi et al. [242] developed a nanosystem (sPDMA@ICG NPs) formed by the self‐assembly of indocyanine green (ICG) and star‐shaped polycationic brushes (sPDMA). This system exhibits excellent adsorption and penetration capabilities against *P. gingivalis*, efficiently delivering ICG to deep periodontal pockets. Upon laser irradiation (808 nm, 2 W/cm²) for 5 min, the system effectively inhibited Pg growth in vitro and reduced alveolar bone resorption and inflammation in vivo, highlighting its clinical translational potential [242]. To address the challenges posed by antibiotic resistance and biofilm formation, which diminish therapeutic efficacy, Ma et al. [243] developed a novel PDT‐driven controlled CO release system (Ce6&CO@FADP). This multifunctional antimicrobial system is safe and efficient, achieving bacterial infection treatment and biofilm eradication in vivo, presenting significant applications in antimicrobial therapies [243]. However, excessive ROS generation during PDT can cause irreversible damage to periodontal tissues. To mitigate this, Sun et al. [244] designed a multifunctional nanocomposite (CeO₂@Ce6) with both antibacterial and anti‐inflammatory properties. This composite material regulates ROS levels and macrophage polarization, avoiding PDT‐induced side effects while achieving sequential “antibacterial first, anti‐inflammatory later” modulation [244]. In summary, the application of nanotechnology has significantly improved the bioavailability and targeting capabilities of photosensitizers, optimized PDT's therapeutic efficacy, and demonstrated considerable advantages in reducing potential side effects.

PDT combined with nanotechnology demonstrates immense potential in the treatment of periodontitis. However, current research predominantly focuses on enhancing its antibacterial efficacy, with limited exploration of its multifunctionality and integrative therapeutic capabilities. Future advancements should aim to address the limitations of conventional photosensitizers by designing nanoplatforms that balance high efficacy with safety, thereby further optimizing the therapeutic performance of PDT. Specifically, by incorporating diverse functional materials, it is possible to develop comprehensive nanotherapeutic platforms that integrate antibacterial PDT, gas therapy, antioxidant treatment, immunomodulation, and tissue repair. Such platforms could effectively target the complex pathological mechanisms of periodontitis while significantly enhancing the specificity and comprehensiveness of treatment, thereby meeting the diverse demands of clinical practice. Simultaneously, future investigations should prioritize PDT's potential in reducing side effects, improving drug delivery efficiency, and enhancing tissue regeneration capabilities, providing more reliable solutions for the precision treatment of periodontitis.

## 6 Conclusions and Future Directions

Maintaining appropriate ROS levels is essential for preserving periodontal tissue homeostasis and creating a conducive environment for tissue regeneration. However, excessive ROS production triggers localized OS, which exacerbates periodontal tissue damage. Reducing ROS overproduction and protecting cells from OS is a viable therapeutic strategy for periodontitis. This review summarizes recent advances in understanding the molecular mechanisms by which ROS drive periodontitis, focusing on:

1. ROS‐induced cell damage through key inflammatory pathways, such as NF‐κB, p38 MAPK, JNK, and ERK; and
2. The activation of OS‐related proteins, like Nrf2 and MAVS, which trigger the NLRP3 inflammasome, leading to tissue destruction. Emerging therapeutic strategies for periodontitis have garnered increasing attention, particularly the use of antioxidants, PDT, and their synergistic application with nanomaterials. These approaches hold promise for enhancing treatment efficacy by targeting ROS‐mediated mechanisms and improving local tissue responses. A deeper understanding of the processes could lead to the development of more effective treatments for periodontitis.

In recent years, antioxidants, PDT and novel nano‐biomaterials have emerged as promising tools for preventing and treating periodontitis. However, several challenges remain:

1. The precise molecular mechanisms linking ROS to periodontitis are not fully understood;
2. The threshold for ROS levels in both physiological and pathological conditions is unclear;
3. There is no consensus on the optimal dosage of ROS scavengers; and
4. Clinical trials evaluating the long‐term efficacy of antioxidants are lacking. These obstacles must be overcome before periodontal tissue engineering can fully achieve its goal of bone regeneration. Given the role of antioxidants in controlling inflammation and promoting tissue repair, there is great interest in developing new materials with anti‐inflammatory, antibacterial, antioxidant, and osteogenic properties. While many antioxidants have shown efficacy in modulating ROS‐related signaling pathways, their potential to target the NLRP3 inflammasome remains underexplored. This could represent a new approach to mitigating periodontal tissue damage.

Future research in the field of ROS and periodontitis should prioritize several areas to enhance our understanding and improve treatment outcomes. First, further investigation is required into the molecular mechanisms by which ROS contribute to periodontitis pathogenesis. While pathways like NF‐κB, MAPK, and NLRP3 have been identified as central mediators of ROS‐induced inflammation and tissue degradation, their interactions and regulatory networks within periodontal tissues remain incompletely understood. Unraveling these relationships could provide new therapeutic targets, especially by blocking specific ROS‐mediated signaling pathways. Second, it is crucial to define the precise ROS levels that balance physiological functions with preventing OS. Identifying these thresholds could inform therapeutic strategies. Third, given the potential of antioxidants, future research should explore the efficacy and safety of antioxidant treatments in periodontal care. Long‐term clinical trials are necessary to assess their impact on periodontal regeneration and inflammation control. Fourth, in light of the potential of photosensitizers, future research should focus on addressing the limitations of traditional photosensitizers, such as their types, concentrations, and the wavelength and intensity of the light source, as well as the availability of oxygen, all of which influence the stability and efficacy of treatment outcomes. Incorporating nanomaterials offers a promising approach to enhance the delivery efficiency of photosensitizers and promote periodontal tissue regeneration. Additionally, emerging antioxidant delivery systems, including nanobiomaterials, present exciting prospects for targeted therapy. These systems are particularly advantageous in modulating ROS levels at inflamed sites, paving the way for synergistic effects when combined with PDT. Extensive clinical trials are still required to validate their safety and efficacy, ensuring their practical application in clinical settings. However, the optimal dosing, timing, and combination of these agents remain to be fully elucidated. Finally, targeting the NLRP3 inflammasome through antioxidant and ROS‐modulating therapies presents a novel avenue for preventing periodontal tissue degradation. Future studies should explore the potential of antioxidants not only as ROS scavengers but also as modulators of NLRP3 activation, aiming to mitigate both inflammation and OS in periodontitis.

## Author Contributions

**Shuyu Yang:** writing – original draft, conceptualization, visualization. **Xi Yang:** writing – review and editing, conceptualization, supervision, funding acquisition.

## Ethics Statement

This study was conducted without the involvement of human participants or animals as subjects.

## Conflicts of Interest

The authors declare no conflicts of interest.

## Data Availability

Data sharing not applicable to this article as no datasets were generated or analysed during the current study.
